# Clinical characteristics and prognosis of amyopathic dermatomyositis patients with interstitial lung disease: insights from a retrospective cohort

**DOI:** 10.1186/s13023-025-03575-w

**Published:** 2025-02-06

**Authors:** Yanan Ying, Tingting Wu, Long Wang, Yun Zhang, Yiming Yu, Zaichun Deng, Qunli Ding

**Affiliations:** 1 Department of Respiratory and Critical Care Medicine, Key Laboratory of Respiratory Disease of Ningbo, The First Affiliated Hospital of Ningbo University, 59 Liuting Street, Haishu District, Ningbo, Zhejiang China; 2 Rheumatology Department, The First Affiliated Hospital of Ningbo University, Ningbo, Zhejiang China

**Keywords:** Amyopathic dermatomyositis, Interstitial lung disease, Nonspecific interstitial pneumonia, Organizing pneumonia, Lactate dehydrogenase

## Abstract

**Introduction:**

The diagnosis of amyopathic dermatomyositis with interstitial lung disease (ADM-ILD) is challenging due to the lack of typical skin features and overlapping syndromes. We aimed to determine the characteristics and prognosis of patients with ADM-ILD to further guide their clinical management.

**Methods:**

A retrospective cohort study comprising 190 Chinese patients diagnosed with interstitial lung disease (ILD) was conducted. Patients were stratified into four groups using the Sontheimer criteria and predominant high-resolution computed tomography (HRCT) patterns. Demographic features, clinical presentation, laboratory parameters, duration of ILD, and follow-up data were analysed.

**Results:**

There were significant differences in the clinical parameters among the 190 patients with ILD in the amyopathic dermatomyositis (ADM, *n* = 69) and control (*n* = 121) groups. The ADM with nonspecific interstitial pneumonia (NSIP) group (*n* = 46) presented increased haemoglobin (125.93 ± 12.91 g/L, *p* = 0.005), creatine kinase-MB (15.19 ± 8.58 U/L, *p* < 0.001), and partial pressure of oxygen (93.08 ± 26.20 mmHg, *p* = 0.003) levels and decreased β2-microglobulin (2.61 ± 1.21 mg/L,* p* = 0.039) levels compared to the control-NSIP group (*n* = 92). The ADM with organizing pneumonia (OP) group (*n* = 23) had a greater percentage of females (7/16, *p* = 0.023) and higher alanine aminotransferase (30.30 ± 20.67 U/L, *p* = 0.039) and aspartate aminotransferase (53.35 ± 65.86 U/L, *p* = 0.003) levels than the control-OP group (*n* = 29). Both the ADM-NSIP and OP groups presented elevated lactate dehydrogenase (LDH) levels (290.61 ± 86.49 U/L,* p* = 0.009; 317.35 ± 181.32 U/L, *p* = 0.003, respectively) and increased anti-nuclear antibody (ANA) positivity rates (82.61%, p = 0.01; 73.91%, *p* < 0.001, respectively). Notably, 81.26% of patients with ADM-NSIP/OP had LDH levels above normal. The serum LDH levels could be used to distinguish patients with ADM-NSIP/OP (sensitivity: 73.91%, specificity: 82.64%). Survival was shorter among patients with ADM-OP than among control patients (*p* = 0.002). Cox multivariate analysis revealed that age (*p* = 0.002), smoking status (*p* = 0.011), anti-melanoma differentiation-associated gene 5 (MDA5) antibody (*p* = 0.017), and white blood cell count (*p* = 0.004) were independent predictors of shorter survival.

**Conclusions:**

Elevated serum LDH levels in patients predominantly presenting with NSIP or OP patterns may indicate the presence of ADM-ILD. The identified prognostic factors underscore the importance of early detection and personalized management strategies for optimizing outcomes in patients with ADM-ILD.

**Supplementary Information:**

The online version contains supplementary material available at 10.1186/s13023-025-03575-w.

## Introduction

Idiopathic inflammatory myopathies (IIMs) [1] are characterized by distinct skin manifestations and exhibit heterogeneity in their pathophysiological features and prognoses. A new classification of IIMs, which is based on clinical manifestations and myositis-specific autoantibodies, involves four subgroups [2]: dermatomyositis (DM), inclusion body myositis (IBM), immune-mediated necrotizing myopathy (IMNM), and antisynthetase syndrome (ASS). This classification is considered superior to that of previous systems. Patients presenting with DM-consistent skin findings but minimal or no evidence of muscle involvement upon examination or diagnostic workup are classified as having amyopathic dermatomyositis (ADM) [3]. ADM is frequently complicated by interstitial lung disease (ILD), with an incidence as high as 90%, which can sometimes lead to severe, life-threatening complications [4, 5]. Notably, rapidly progressive ILD (RPILD) has emerged as a significant concern, with a fatality rate of 50% among patients with dermatomyositis within six months [6, 7]. The high mortality associated with RPILD is largely due to its resistance to standard combination immunosuppressive therapies involving high-dose glucocorticoids, cyclophosphamide, and calcineurin inhibitors [8, 9]. Therefore, the early detection of ADM-ILD is critical, as prompt intervention significantly improves patient prognosis [10].

In clinical practice, approximately 20% of patients with DM lack classic muscle manifestations and present with normal serum creatine kinase (CK) levels [11, 12]. Currently, there are no standardized diagnostic criteria for ADM, and different studies use various definitions. According to the literature, 25% of patients with ADM are misdiagnosed due to the absence of two of three typical skin features, often leading to misclassification as lupus erythematosus [13]. Therefore, diagnosing ADM can be particularly challenging without distinct dermatologic features. For respiratory specialists, high-resolution computed tomography (HRCT) is indispensable in diagnosing and assessing interstitial lung disease. On the basis of imaging and histopathology, IIM-associated ILD is difficult to distinguish from idiopathic forms of interstitial pneumonia. Various cohort studies regarding IIM-associated ILD have reported predominantly nonspecific interstitial pneumonia (NSIP), organizing pneumonia (OP), or a combination of NSIP/OP imaging patterns, often characterized by ground‒glass opacities, reticulation, and consolidation [14–16]. In contrast, usual interstitial pneumonia (UIP) and diffuse alveolar damage (DAD) patterns are uncommon.

As ADM-ILD frequently presents with NSIP, OP, or a mixed pattern and often lacks classic muscle manifestations and elevated CK levels, there is a compelling need to identify specific characteristics that can distinguish ADM from other diseases with similar patterns. Therefore, we designed a cohort consisting of patients who primarily displayed NSIP or OP patterns to further evaluate the distinguishing characteristics of ADM. This approach aims to increase the effectiveness of screening for myositis-specific antibodies (MSAs) and myositis-associated antibodies (MAAs) in potential patients.

## Methods

### Subjects

We retrospectively reviewed the medical records of patients who were diagnosed with interstitial lung disease (*n* = 382) between January 2017 and December 2022 at the Ningbo University Affiliated First Hospital. According to the ATS/ERS consensus criteria [17], radiological patterns on HRCT at the initial presentation were verified by two experienced radiologists who were blinded to the clinical data (categorized as NSIP, OP, and others). We classified the patients into two cohorts on the basis of their predominant radiographic pattern: the NSIP group and the OP group. The diagnoses of all patients, supported by comprehensive clinical data, involved multidisciplinary discussions with experienced pulmonologists specializing in interstitial lung diseases and pathologists.

In accordance with the Sontheimer criteria [18], 69 patients in our study were identified as having ADM-ILD. The exclusion criteria were as follows: (1) patients with idiopathic inflammatory myopathy who had elevated CK and/or muscular manifestations (*n* = 19); (2) patients with malignancy at the beginning of diagnosis (*n* = 10); (3) patients with a combination of 2 or more connective tissue diseases (*n* = 4); (4) patients treated with glucocorticoids or immunosuppressive therapy and other related treatments prior to data collection (*n* = 15); and (5) patients with a history of the use of drugs that cause hallmark cutaneous manifestations of connective tissue disease (CTD) (*n* = 2).

**Fig. 1:**
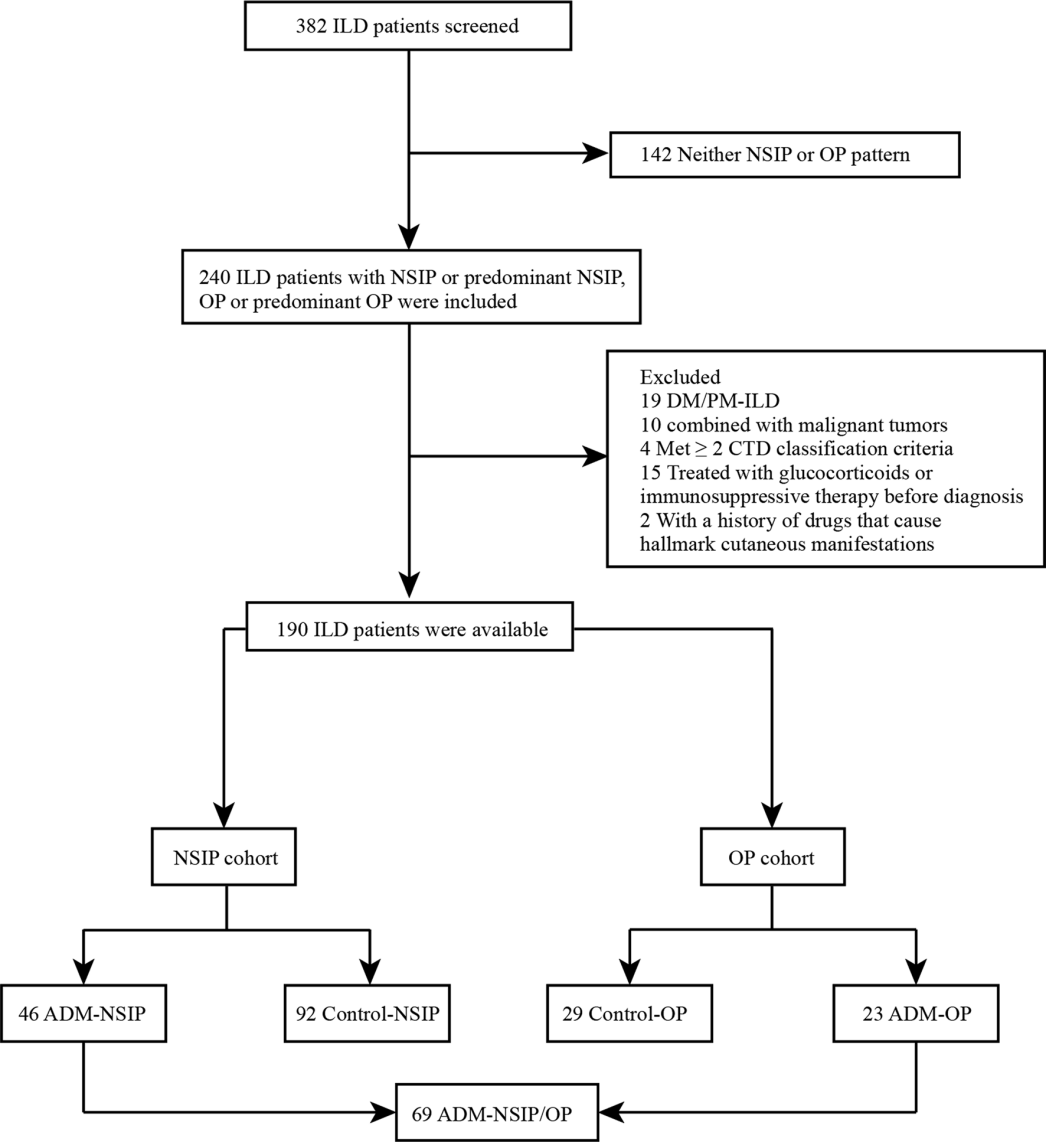
Flow chart of the study. ILD: interstitial lung disease; NSIP: nonspecific interstitial pneumonia; OP: organizing pneumonia; DM/PM: dermatomyositis/polymyositis; CTD: connective tissue disease; ADM: amyopathic dermatomyositis

Specifically, our ILD cohort included 190 patients who were subdivided into 4 groups. Patients with NSIP or predominant NSIP were assigned to the ADM group (*n* = 46) and NSIP control group (*n* = 92), including 14 patients with rheumatoid arthritis (RA), 19 with primary Sjogren’s syndrome (pSS), 12 with systemic sclerosis (SSc), 8 with systemic lupus erythematosus (SLE), 3 with mixed connective tissue disease (MCTD), 15 with idiopathic interstitial pneumonia (IIP), 19 with microscopic polyangiitis (MPA), and 12 with interstitial pneumonia with autoimmune features (IPAF). Similarly, patients with OP or predominant OP were included in the ADM group (*n* = 23) and OP control group (*n* = 29), including 24 with cryptogenic organizing pneumonia (COP), 3 with RA, 1 with IPAF, and 1 with SLE. The flow chart of this study design is shown in Fig. 1. This cohort study was approved by the institutional review board of The First Affiliated Hospital of Ningbo University (IRB no: KS20233005, retrospectively registered).

### Data collection

Clinical data, including patient history, clinical presentation, laboratory results, detailed clinicopathological information, and chest HRCT images, were obtained by reviewing the medical records at the time of diagnosis. The follow-up time began when ILD was observed in patients and ended on December 31, 2022. Survival status was determined via a review of the medical records or telephone calls. We recorded the age, sex, and smoking history of each patient as demographic features. The clinical presentations included rash, especially the hallmark cutaneous manifestations of DM, such as a heliotrope rash and Gottron sign, and minor cutaneous manifestations, including cutaneous ulcers, mechanic hands, poikiloderma, V-signs and the Holster sign. Systemic symptoms included noninfectious fevers, Raynaud’s phenomenon, arthralgia and/or arthritis, and weight loss or fatigue.

The serum white blood cell count; absolute lymphocyte count; haemoglobin level; platelet count; C-reactive protein level; erythrocyte sedimentation rate; and the levels of albumin, alanine aminotransferase, aspartate aminotransferase, creatine kinase, creatine kinase-MB, lactate dehydrogenase, and β2 microglobulin were detected. Immunological parameters, including antinuclear antibodies, myositis-specific antibodies, and myositis-associated antibodies, were recorded. Blood gas indicators included arterial oxygen partial pressure, the oxygenation index (OI), and arterial lactate levels. Pulmonary function tests, including measurements of the diffusing capacity of the lung for carbon monoxide (DLco), forced expiratory volume in one second (FEV1), and forced vital capacity (FVC), were performed in some patients. The results are expressed as percentages of the normal predicted values. All data for these variables were obtained before the initiation of treatment to assess the predictive factors for ADM-ILD.

### Statistical analysis

The data are presented as the frequency and percentage for categorical variables and mean ± standard deviation (SD) for numeric variables. To compare the differences in study participants’ characteristics across the groups, we used a chi-square test or Fisher’s exact test for categorical variables and an independent test or Mann‒Whitney U test for continuous variables, depending on the distribution of the data. We performed receiver operating characteristic (ROC) curve analysis to assess the sensitivity and specificity of LDH and ANA for distinguishing ADM-NSIP/OP. We also performed Kaplan–Meier survival analysis to estimate the survival rates, plotted the survival curves, and used the log-rank test to compare the differences between groups. We used univariate and multivariate Cox proportional hazards regression analyses to identify predictors of ADM-ILD-related death. All statistical analyses were carried out using SPSS 24.0 (SPSS Inc., Chicago, IL, USA) and GraphPad Prism 9 software (GraphPad Software, San Diego, CA, USA). A* p* value less than 0.05 was used to indicate statistical significance.

## Results

### Comparison of clinical parameters between the ADM-NSIP/OP and control groups

The baseline clinical characteristics of the 69 patients in the ADM-NSIP/OP group are summarized in Table 1. In the cohort, the patients had a mean age of 58.12 ± 13.11 years and a female predominance (26/43). Gottron’s sign (37.68%) and heliotrope rash (43.48%) were common in these patients, especially in patients with ADM-OP. Minor cutaneous manifestations were present in 34.78% of the patients. The most prevalent myositis-specific antibody (MSA) detected was anti-MDA5 (24.64%), with the ADM-OP group showing a greater proportion (47.83%). In the ADM-NSIP group, anti-PL-7 (26.09%) and anti-Jo-1 (21.74%) antibodies were more common. Anti-RO-52 emerged as the most prevalent myositis-associated antibody (MAA), detected in 33 patients (47.83%), with a higher positivity rate in the ADM-NSIP group (54.35%).

**Table 1:** Baseline clinical characteristics of patients in the ADM-NSIP/OP groups

Characteristics	ALL patients (*n* = 69)	ADM-NSIP (*n* = 46)	ADM-OP (*n* = 23)
Age	58.12 ± 13.11	58.35 ± 12.08	57.65 ± 15.26
Sex (M/F)	26/43	19/27	7/16
Dermatological features			
Heliotrope rash, *n*	30, (43.48%)	17, (36.96%)	13, (56.52%)
Gottron’s sign, *n*	26, (37.68%)	10, (21.74%)	16, (69.57%)
Minor cutaneous manifestations, *n*	24, (34.78%)	16, (34.78%)	7, (30.43%)
Myositis autoantibodies			
MSAs			
Anti-MDA5	17 (24.64%)	6, (13.04%)	11, (47.83%)
Anti-PL-7	14 (20.29%)	12, (26.09%)	2, (8.70%)
Anti-Jo-1	13 (18.84%)	10, (21.74%)	3, (13.04%)
Anti-PL-12	8 (11.59%)	6, (13.04%)	2, (8.70%)
Anti-EJ	8 (11.59%)	5, (10.87%)	3, (13.04%)
Anti-Mi-2	3 (4.35%)	3, (6.52%)	0, (0%)
Anti-SRP	2 (2.90%)	0, (0%)	2, (8.70%)
MAAs			
Anti-RO-52	33 (47.83%)	25, (54.35%)	8, (34.78%)
Anti-PM-Scl 75	4 (5.80%)	3, (6.52%)	1, (4.35%)
Anti-SS-A	3 (4.35%)	3, (6.52%)	0, (0%)
Anti-PM-Scl 100	2 (2.90%)	1, (1.45%)	1, (4.35%)
Anti-Ku	2 (2.90%)	1, (1.45%)	1, (4.35%)
Anti-U1-RNP	1 (1.45%)	1, (1.45%)	0, (0%)

ADM: amyopathic dermatomyositis; M: male; F: female; NSIP: nonspecific interstitial pneumonia; OP: organized pneumonia; Minor cutaneous manifestations: 9 Mechanic hands, 6 V-sign, 4 Cutaneous ulcers, 3 Poikiloderma, 2 Holster sign; MSA: myositis-specific autoantibody; MAA: myositis-associated autoantibody; MDA5: melanoma differentiation-associated gene 5; Jo-1: histidyl-tRNA synthetase; PL-7: threonyl-tRNA synthetase; PL-12: alanyl-tRNA synthetase; EJ: glycyl-transfer ribonucleic acid synthetase; Mi-2: nuclear matrix protein 2; SRP: signal recognition particle; RO-52: Ro/SSA 52 kD; PM-Scl: polymyositis-scleroderma; SS-A: Sjögren’s syndrome-related antigen A; RNP: Ribonucleoprotein

We compared the clinical data of the ADM-NSIP/OP groups and their respective control groups, which exhibited similar radiological patterns. The demographic and main characteristics of the patients with NSIP and OP are summarized in Table 2. As shown in the table, there were some differences between the groups in terms of demographic features and haematological, biochemical, and immunological data. The comparison between the two groups revealed similar ages at onset, with mean ages of approximately middle to late adulthood. However, significant sex disparities were observed within the OP group (*p* = 0.023), characterized by a male-to-female ratio of 7:16. Among the peripheral blood parameters, significant differences were observed in the levels of HB (125.93 ± 12.91 vs. 123.49 ± 20.12 g/L, *p* = 0.005), CK-MB (15.19 ± 8.58 vs. 11.15 ± 4.97 U/L, *p* < 0.001), and β2-MG (2.61 ± 1.21 vs. 3.38 ± 2.47 mg/L, *p* = 0.039) within the NSIP groups, whereas the alanine transaminase (30.30 ± 20.67 vs. 21.24 ± 14.14 U/L, *p* = 0.039) and aspartate transaminase (53.35 ± 65.86 vs. 23.83 ± 11.16 U/L, *p* = 0.003) levels varied significantly within the OP groups. Notably, both the ADM-NSIP and ADM-OP groups presented markedly greater lactate dehydrogenase (LDH) levels than did the control group (290.61 ± 86.49 U/L vs. 207.04 ± 56.64 U/L, *p* = 0.009; 317.35 ± 181.32 U/L vs. 185.10 ± 36.97 U/L, *p* = 0.003, respectively). Additionally, patients in the ADM-NSIP/OP group had a greater frequency of positive antinuclear antibody tests than did patients in the control group (82.61% vs. 60.87%, *p* = 0.010; 73.91% vs. 17.24%, *p* < 0.001). Regrettably, not all patients with ILD underwent blood gas analysis and pulmonary function tests as those results were not documented in the medical records. The number of cases and specific statistical data are reported in Table 2. On the basis of these limited data, patients with ADM-NSIP/OP may not initially present with more severe respiratory impairment than control patients at the time of diagnosis. Although there were trends suggestive of decreased overall diffuse capacity, the differences observed did not appear to be statistically significant (*p* = 0.390, *p* = 0.111, respectively).

**Table 2:** Comparison of clinical data between the ADM-NSIP/OP group and the corresponding control group

	ADM-NSIP (*n* = 46)	Control-NSIP (*n* = 92)	*P*	ADM-OP (*n* = 23)	Control-OP (*n* = 29)	*P*
*Demographic feature*						
Age (years)	58.35 ± 12.08	65.22 ± 14.34	0.310	57.65 ± 15.26	62.76 ± 15.16	0.838
Sex (M/F)	19/27	28/64	0.204	**7/16**	**18/11**	**0.023**
Smoking history, *n* (%)	8 (17.39%)	25 (27.17%)	0.122	4 (17.39%)	11 (37.93%)	0.104
Systemic symptoms, *n* (%)	20 (43.48%)	56 (60.87%)	0.053	8 (34.78%)	7 (24.14%)	0.400
*Peripheral blood*						
WBC (× 10^9^/L)	6.92 ± 3.37	7.04 ± 3.43	0.494	6.46 ± 3.69	7.94 ± 2.50	0.057
Lymphocyte (× 10^9^/L)	1.32 ± 0.64	1.56 ± 0.59	0.260	1.12 ± 0.65	1.52 ± 1.15	0.664
HB (g/L)	**125.93 ± 12.91**	**123.49 ± 20.12**	**0.005**	120.57 ± 17.38	124.34 ± 20.01	0.538
PLT (× 10^9^/L)	246.72 ± 73.28	227.11 ± 85.48	0.390	225.74 ± 73.60	279.66 ± 99.40	0.187
hsCRP (mg/L)	15.78 ± 32.30	20.22 ± 34.25	0.187	18.55 ± 31.52	48.08 ± 42.67	0.096
ESR (mm/h)	41.20 ± 33.49	41.16 ± 34.69	0.589	37.95 ± 25.50	58.93 ± 35.40	0.087
ALB (g/L)	36.41 ± 4.98	37.44 ± 5.06	0.848	33.37 ± 5.03	36.06 ± 5.40	0.953
ALT (U/L)	30.33 ± 34.28	22.77 ± 17.07	0.120	**30.30 ± 20.67**	**21.24 ± 14.14**	**0.039**
AST (U/L)	35.35 ± 33.01	30.16 ± 16.94	0.052	**53.35 ± 65.86**	**23.83 ± 11.16**	**0.003**
CK (U/L)	74.40 ± 39.77	83.27 ± 105.39	0.122	83.45 ± 50.96	72.48 ± 66.81	0.288
CK-MB (U/L)	**15.19 ± 8.58**	**11.15 ± 4.97**	** < 0.001**	13.38 ± 8.73	11.28 ± 7.47	0.068
LDH (U/L)	**290.61 ± 86.49**	**207.04 ± 56.64**	**0.009**	**317.35 ± 181.32**	**185.10 ± 36.97**	**0.003**
β2-MG (mg/L)	**2.61 ± 1.21**	**3.38 ± 2.47**	**0.039**	2.94 ± 1.61	2.34 ± 0.74	0.096
*Immunological parameters*						
ANA (1 ≥ 80), *n* (%)	**38 (82.61%)**	**56 (60.87%)**	**0.010**	**17 (73.91%)**	**5 (17.24%)**	** < 0.001**
Arterial blood gas	*n* = 39	*n* = 59		*n* = 17	*n* = 21	
PaO_2_ (mmHg)	**93.08 ± 26.20**	**80.24 ± 16.07**	**0.003**	82.05 ± 22.10	75.19 ± 16.68	0.807
OI (mmHg)	401.75 ± 76.34	372.28 ± 81.85	0.666	346.37 ± 86.39	328.25 ± 93.01	0.755
Lactate (mmol/L)	1.62 ± 0.69	1.74 ± 0.86	0.813	1.81 ± 1.00	1.74 ± 0.81	0.935
Pulmonary function tests	*n* = 29	*n* = 52		*n* = 14	*n* = 9	
FEV1, %predicted	76.28 ± 17.84	88.56 ± 18.38	0.998	74.16 ± 19.69	85.27 ± 27.57	0.281
FVC, %predicted	75.32 ± 19.06	83.07 ± 16.01	0.428	76.88 ± 17.27	78.01 ± 21.46	0.557
DLco, %predicted	53.94 ± 16.77	64.21 ± 20.10	0.390	54.21 ± 15.70	71.28 ± 9.70	0.111

The bold value indicates that the *p*-value for that particular comparison is < 0.05, meaning there is a statistically significant difference between the groups being compared

The data are presented as the mean (M) ± standard deviation (SD), and *p* < 0.05 was considered to indicate statistical significance

ADM: amyopathic dermatomyositis; NSIP: nonspecific interstitial pneumonia; OP: organized pneumonia; M: male; F: female; WBC: white blood cell; HB: haemoglobin; PLT: platelet; hsCRP: high-sensitivity C-reactive protein; ESR: erythrocyte sedimentation rate; ALB: albumin; ALT: alanine aminotransferase; AST: aspartate aminotransferase; CK: creatine kinase; CK-MB: creatine kinase and its MB isoenzyme; LDH: lactate dehydrogenase; β2-MG: beta2-microglobin; ANA: antinuclear antibodies; PaO_2_: arterial oxygen partial pressure; OI: oxygenation index; FEV1: forced expiratory volume in 1 s; FVC: forced vital capacity; DLco: diffusing capacity of the lung for carbon monoxide

### Diagnostic value of serum LDH and ANA in the ADM-NSIP/OP groups

**Fig. 2:**
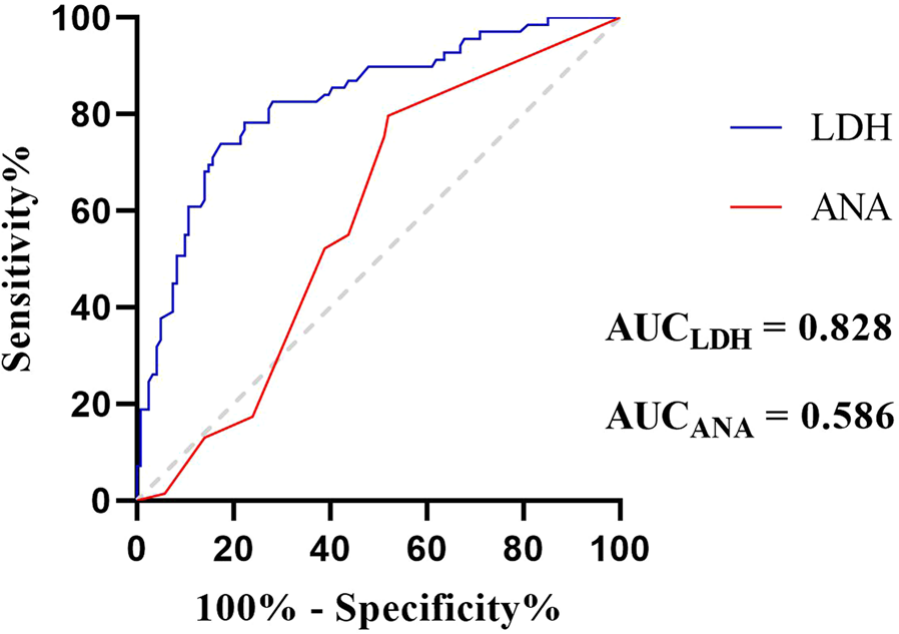
ROC curve analysis of the serum LDH levels and ANA positivity

In the comparative descriptive analysis, significantly elevated serum LDH levels were observed in both the NSIP and OP groups compared with their respective controls. Furthermore, within the ADM-NSIP/OP patient cohort, 81.26% (56/69) patients exhibited LDH levels that surpassed the normal range of 106–211 U/L, with values ranging from a minimum of 154 U/L to a maximum of 1043 U/L. To assess the diagnostic performance of serum LDH for the discrimination of ADM-NSIP/OP, receiver operating characteristic curve analysis was performed and yielded a cutoff of 235.5 U/L. At this threshold, the serum LDH level had a sensitivity of 73.91% and a specificity of 82.64% in discriminating the ADM-NSIP/OP group (area under the curve (AUC) = 0.828; 95% CI, 0.7658–0.8895;* p* < 0.001). The positive and negative predictive values were 60.22% (95% CI, 50.05–69.57%) and 86.60% (95% CI, 78.41–92.00%), respectively. However, ANA positivity did not have diagnostic utility (AUC = 0.586; 95% CI, 0.5043–0.6666; *p* = 0.05) (Fig. 2).

### Survival in the NSIP/OP groups

**Fig. 3:**
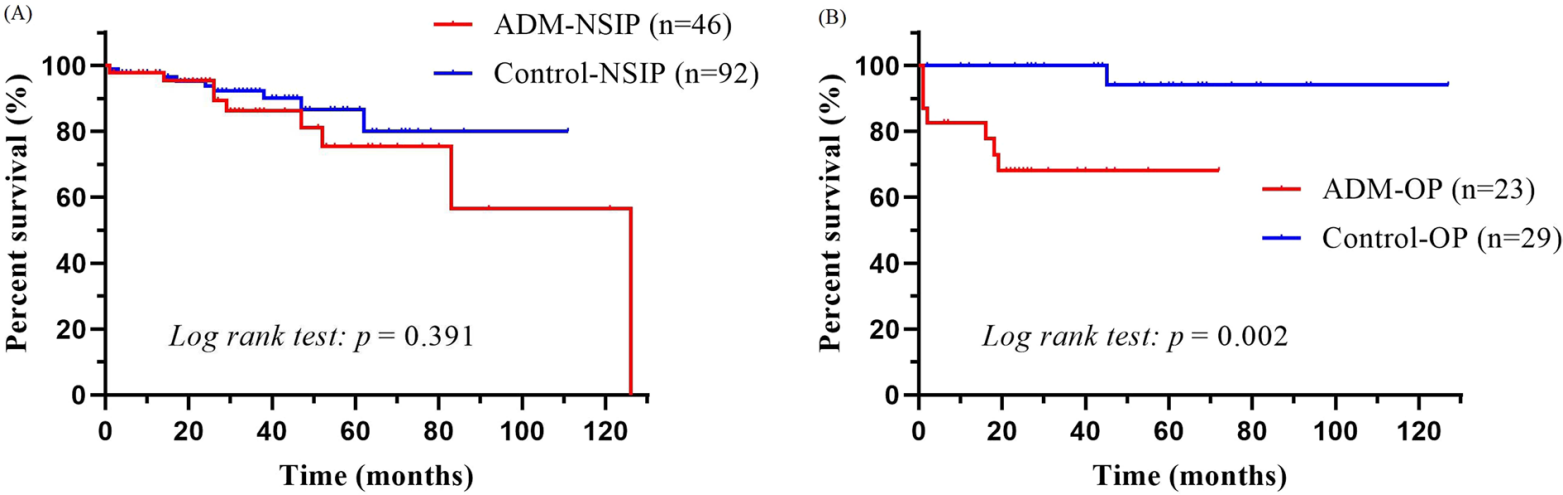
**A** Kaplan–Meier survival curves of patients with predominant nonspecific interstitial pneumonia (NSIP). There was no significant difference in the survival curves between the amyopathic dermatomyositis (ADM) group and the control group (log-rank test, *p* = 0.391). **B** Kaplan–Meier survival curves of patients with predominant organizing pneumonia (OP). Patients with ADM-OP had a significantly lower survival rate than did patients in the OP control group (log-rank test, *p* = 0.002)

In our cohort, the median follow-up time of the 69 patients with ADM was 33 months, and all-cause mortality was observed in 16 (23.19%) patients during follow-up. Five patients died of RP-ILD within a 1–2-month period, with one classified in the NSIP group and the remaining four in the OP group. We evaluated the survival outcomes of patients in the ADM-NSIP/OP groups and compared them with those of patients with other clinical subtypes of NSIP or OP. For patients in the ADM-NSIP group, the 1-, 3-, and 5-year overall survival rates were 97.83%, 86.23%, and 75.36%, respectively. Similarly, 97.83%, 92.26%, and 86.69% of the control-NSIP patients survived at 1, 3, and 5 years, respectively. Notably, the Kaplan‒Meier survival curves of the two groups were not significantly different (log-rank test, *p* = 0.391) (Fig. 3A). However, patients with ADM-OP had significantly lower overall survival rates at 1, 3, and 5 years (82.61%, 68.03%, and 68.03%, respectively) than did the control-OP patients (100%, 100%, and 94.12%, respectively), and Kaplan‒Meier survival curves were significantly different between the two groups (log-rank test, *p* = 0.002) (Fig. 3B).

**Fig. 4:**
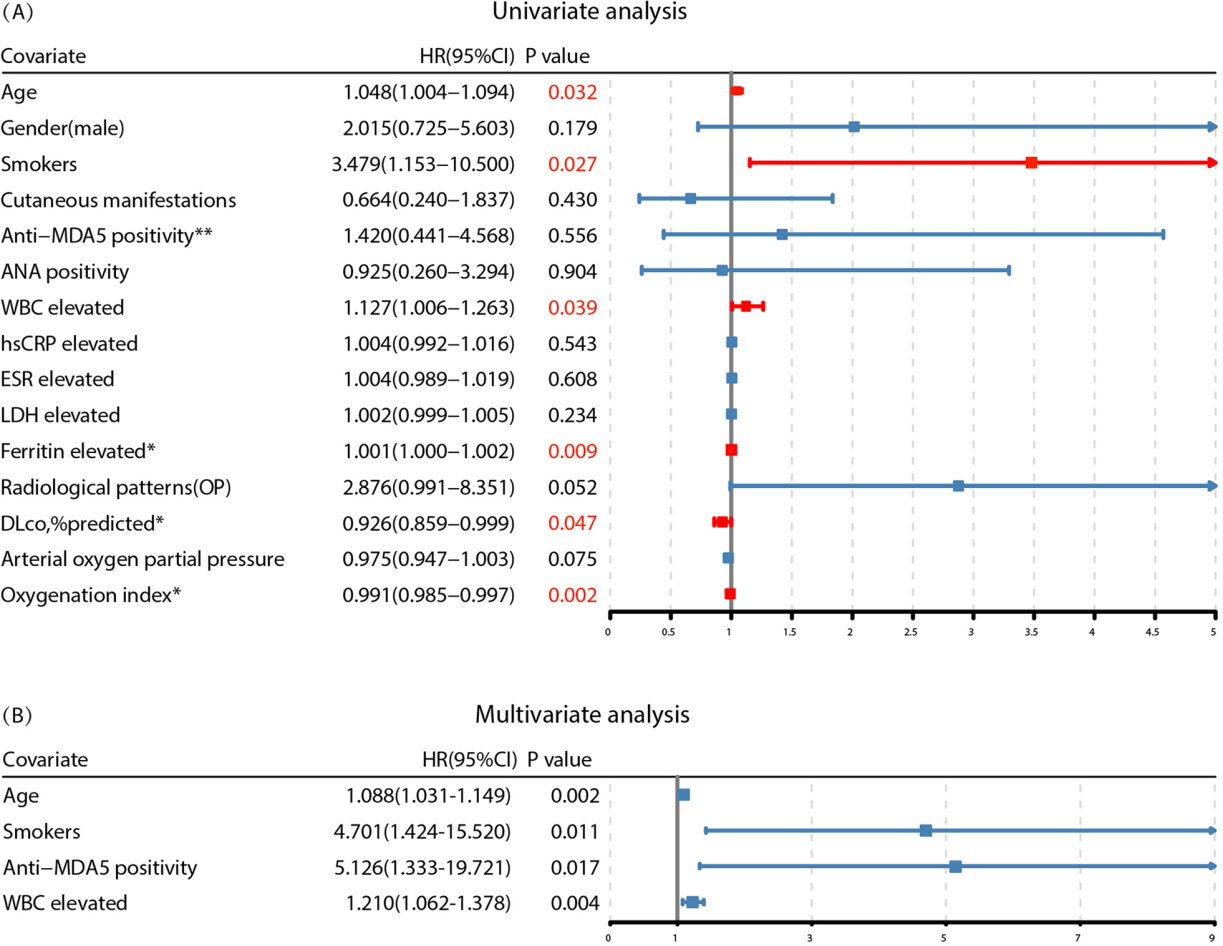
Forest plot of univariate **A** and multivariate **B** Cox regression analyses for the risk factors for all-cause mortality in 69 patients in the ADM-NSIP/OP groups. *p* < 0.05 was considered significant. *Due to insufficient data, ferritin, DLco and oxygenation indices were not included in the multivariate Cox regression model. ** Anti-MDA5 antibody, although not significant in univariate Cox analysis, is widely recognized as an important prognostic marker in myositis-associated ILD and, therefore, was included in the multivariate model

Univariate and multivariate analyses of the risk of all-cause mortality in patients in the ADM-NSIP/OP groups are summarized in Figs. 4A and 4B. Several prognostic factors were identified via univariate analysis. Older age, a history of smoking, a higher WBC and ferritin level, a lower DLco, and a lower oxygenation index (OI) were associate with an increased risk of mortality. Due to the largely missing ferritin, DLco, and OI data, these covariates were not included in the multivariate Cox analysis. According to the multivariate Cox analysis, age [HR = 1.09, 95% CI = 1.03–1.15, *p* = 0.002], a history of smoking [HR = 4.70, 95% CI = 1.42–15.52, *p* = 0.011], anti-MDA5 positivity [HR = 5.13, 95% CI = 1.33–19.72, *p* = 0.017], and white blood cell count [HR = 1.21, 95% CI = 1.06–1.38, *p* = 0.004] remained significant factors that were independently associated with shorter survival.

## Discussion

In our retrospective study, we aimed to analyse clinical features to underscore the pivotal role of an early ADM-ILD diagnosis by comparing 69 patients with ADM-NSIP/OP and 121 ILD controls exhibiting similar radiological patterns. Our findings revealed differences in the levels of HB, CK-MB, β2-MG, and PaO_2_ between the ADM-NSIP and control-NSIP groups, whereas differences in sex and the levels of ALT and AST were detected between the ADM-OP and control-OP groups. Notably, both patients with ADM-NSIP and those with OP presented significantly greater LDH levels than control patients, and there was a high prevalence of ANA positivity. Our analysis further suggested that serum LDH levels demonstrate diagnostic potential for patients with ADM displaying predominant NSIP or OP patterns. Patients with ADM-OP had significantly shorter survival outcomes than did control patients. Age, smoking status, anti-MDA5, and WBC count were found to be independently associated with shorter survival.

Due to the absence of muscle weakness and elevated muscle enzymes, ADM-ILD presents with a range of symptoms, making it difficult to identify. To address this challenge, particularly given the unfamiliarity of nonrheumatologists with dermatological manifestations, we aimed to mitigate the risk of missed diagnoses or misdiagnoses of ADM-ILD. Several laboratory parameters varied in the ADM-ILD cohort, with more than 80% of the patients with ADM-ILD exhibiting significantly elevated LDH levels beyond the normal range compared with those in the control group. In the context of patients with ILD presenting with NSIP and/or OP morphological patterns, our analysis further revealed that serum LDH levels demonstrate diagnostic potential (AUC = 0.828) for ADM. The high negative predictive value (86.60%) suggests that LDH could serve as a valuable screening parameter, with utility in excluding ADM-NSIP/OP during initial patient assessments. Although serum LDH can be elevated under various conditions [19], the elevation of LDH in ADM may suggest several possibilities. First, it could indicate muscle inflammation without the presence of weakness. Patients with DM/PM-ILD often exhibit significant increases in CK and LDH levels. After treatment, CK levels typically decrease to the normal range, and muscle weakness symptoms improve; however, LDH levels remain elevated without decreasing, similar to the conditions found in ADM [20]. Second, the increase in LDH may be related to ILD. A previous study revealed that idiopathic pulmonary fibrosis (IPF) can lead to elevated LDH levels [21], suggesting that the increase in LDH observed in some patients with ADM-ILD may be associated with the presence of pulmonary fibrosis. The specific association between positive ANA titres and ADM-ILD has not been clearly addressed in the literature, and the higher ANA positivity observed in patients with ADM-ILD may be related to the composition of ILD included in the control group, such as COP and IIP. Further large-scale research is needed to validate these findings.

IIMs primarily affect individuals in late middle age and are more common in women [22, 23]. Our study corroborates this demographic trend in patients with ADM, which is particularly evident in patients with the OP pattern. The characteristic dermatological manifestations of DM were observed in the majority of our patients, which underlines their clinical relevance and substantiates the diagnosis of ADM-ILD [24]. MSAs and MAAs are pivotal for understanding the immunological underpinnings of ADM, and different autoantibodies are associated with different clinical features [25]. Anti-MDA5 is a significant autoantibody in dermatomyositis and is linked to distinctive clinical features such as cutaneous ulceration and ILD [26, 27]. Moreover, it is associated with poor prognosis and a high incidence of acute/subacute interstitial pneumonia [6, 28–31], which often requires intensive combined immunosuppressive therapy [9]. In our cohort, anti-MDA5 and anti-RO-52 were the most prevalent autoantibodies and were detected in 72.47% of patients with ADM. Although the anti-MDA5 antibody did not exhibit significant value in the univariate Cox analysis, its significance as a prognostic marker in myositis-associated ILD is widely acknowledged. After inclusion in the multivariate analysis, the highest hazard ratio was demonstrated to be an independent predictor of mortality, with a 5.1-fold increase in risk. Interestingly, we also found that most patients with ADM-ILD with anti-MDA5 positivity exhibited a predominant pattern of OP (11/17 patients). The survival outcomes of patients with ADM-OP significantly differed from those of control patients, with markedly lower survival rates. This observation aligns with studies that reported a high incidence of OP patterns on HRCT in patients with IIM-associated RP-ILD [32, 33]. Taken together, these findings underscore the necessity for close monitoring and potentially more aggressive management strategies in patients with anti-MDA5-positive ADM-OP.

In terms of the prognosis of patients with myositis-associated ILD, recent studies have focused primarily on exploring risk factors for patients with anti-MDA5-positive DM [9, 34, 35], with limited attention to the prognosis of patients with ADM-ILD. Age [36, 37], ferritin level [38, 39], DLco, and OI [40] have been identified as factors linked to poor outcomes by various studies; smoking has been shown to be significantly associated with the incidence of ILD in patients with myositis [41]. With respect to LDH, previous studies have indicated an association between elevated LDH levels and poor prognosis [42, 43]. However, in our study, elevated LDH levels did not significantly impact the prognosis of patients with ADM-ILD. We hypothesize that our patient selection, predominantly from respiratory and rheumatology departments, may have introduced a bias towards milder forms of the disease, potentially masking the effects of LDH on prognosis. To our knowledge, this is the first study to identify an elevated WBC count as an independent predictor of all-cause mortality in patients with ADM-ILD. The WBC count is a nonspecific marker of inflammation and infection, which are recognized causes of acute exacerbation (AE) in patients with ILD. AE is associated with poor prognosis and high mortality, regardless of the underlying type of ILD [44]. Notably, a retrospective longitudinal study of Chinese patients with IIM-ILD reported that infection was the leading cause of death, accounting for 49.3% of all mortality cases [45]. These findings underscore the importance of promptly addressing any signs of infection and improving survival outcomes in patients with ADM-ILD.

The present study is subject to certain potential limitations inherent to its retrospective design. A major limitation is the heterogeneity of our control groups, which included patients with diverse aetiologies of NSIP and OP, potentially introducing bias in our comparative analyses. Additionally, the relatively low number of mortality cases in this study may limit the generalizability of our findings to more severe presentations of the condition. Several studies have underscored the importance of quantitative chest CT scans [46] and the ILD-GAP score [47] in evaluating the severity and extent of ILD. However, we were unable to obtain these data, and the absence of comprehensive pulmonary function tests and blood gas analyses further contributes to the limitations of our study. Consequently, the potential influence of more severe lung injury on the observed increase in LDH levels in patients with ADM-ILD cannot be fully addressed. Notably, indicators such as Krebs von den Lungen-6 (KL-6) and interleukin (IL) levels, which are strongly associated with ILD prognosis, were not included in our study. Moreover, we did not examine the pathological features or genetic susceptibility of patients with ADM-ILD. Therefore, a prospective study with a substantially larger sample size and a randomized controlled trial are needed to further validate our findings.

## Conclusion

In conclusion, our study underscores the potential diagnostic significance of unexplained elevations in LDH levels among patients predominantly displaying NSIP or OP patterns, suggesting the possibility of ADM-ILD. Patients with ADM-OP had shorter survival outcomes. Older age, smoking history, anti-MDA5 positivity, and WBC count were identified as factors associated with increased mortality risk. These findings underscore the importance of early detection and phenotyping in the management of ADM-ILD.

## Supplementary Information

Additional file 1.

## Data Availability

The datasets used in this study are not publicly available due to privacy concerns. However, interested individuals can request access to the data from the corresponding author, which is subject to approval by The First Affiliated Hospital of Ningbo University.
