# Supplementary material for: Clinical characteristics and prognosis of amyopathic dermatomyositis patients with interstitial lung disease: insights from a retrospective cohort
Source: Orphanet J Rare Dis. 2025 Feb 6;20:53. doi: 10.1186/s13023-025-03575-w (PMC11804100; doi:10.1186/s13023-025-03575-w)

**Supplement 1** Kaplan–Meier survival curves of patients with ADM-NSIP/OP. There was a significant difference in the survival curves between the ADM-NSIP and ADM-OP groups (log-rank test, *p*=0.043).


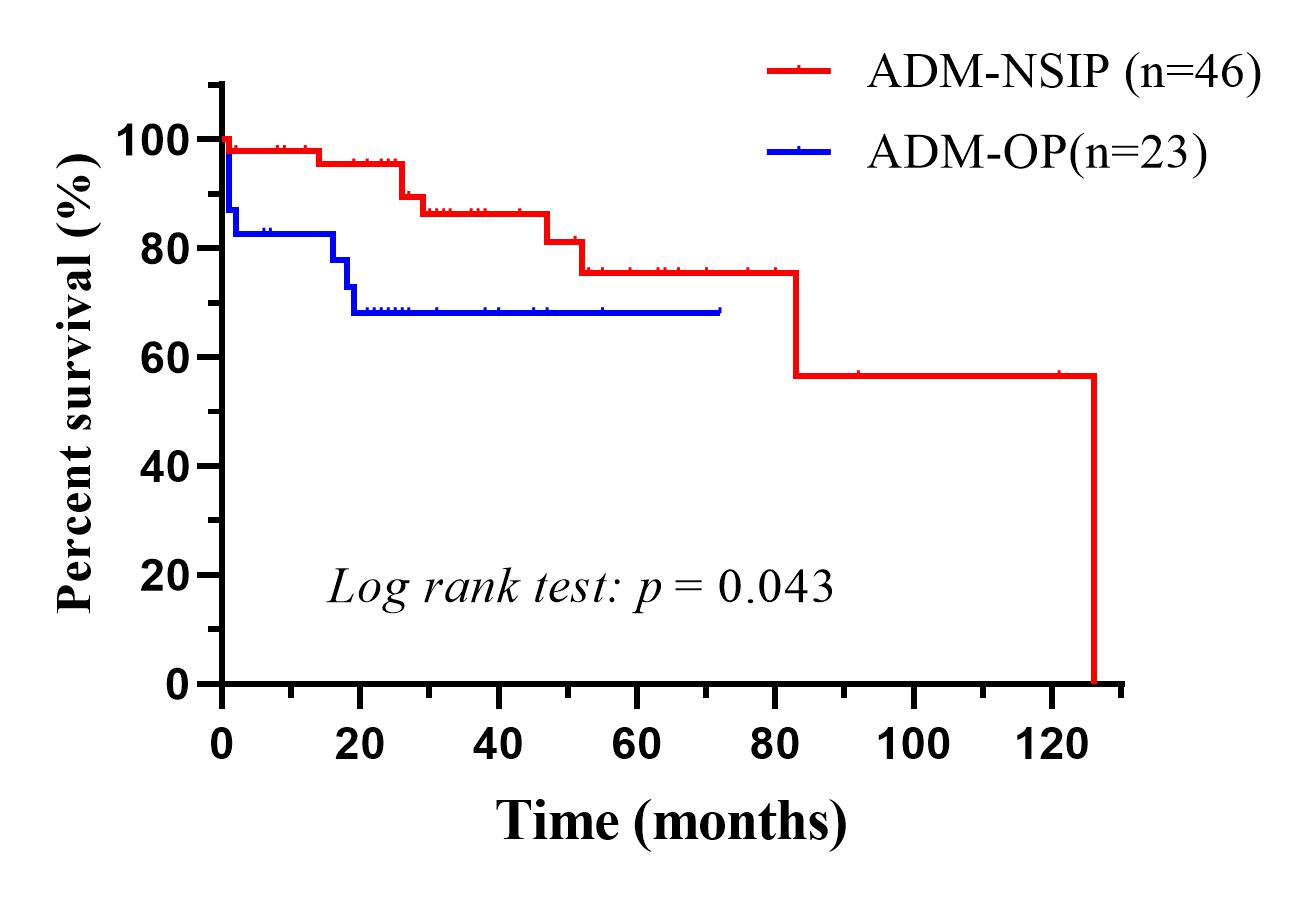

Supplement: Supplementary file 1 — Additional file 1. [file 13023_2025_3575_MOESM1_ESM.docx]
